# Recent advances in understanding and prevention of sudden cardiac death

**DOI:** 10.12688/f1000research.11855.1

**Published:** 2017-08-31

**Authors:** Jamie I. Vandenberg, Matthew D. Perry, Adam P. Hill

**Affiliations:** 1 St Vincent’s Clinical School, University of New South Wales, Darlinghurst, Australia; 2 Victor Chang Cardiac Research Institute, Darlinghurst, Australia

**Keywords:** sudden cardiac death, heart disease, cardiac arrhythmogenesis, genetic heart diseases

## Abstract

There have been tremendous advances in the diagnosis and treatment of heart disease over the last 50 years. Nevertheless, it remains the number one cause of death. About half of heart-related deaths occur suddenly, and in about half of these cases the person was unaware that they had underlying heart disease. Genetic heart disease accounts for only approximately 2% of sudden cardiac deaths, but as it typically occurs in younger people it has been a particular focus of activity in our quest to not only understand the underlying mechanisms of cardiac arrhythmogenesis but also develop better strategies for earlier detection and prevention. In this brief review, we will highlight trends in the recent literature focused on sudden cardiac death in genetic heart diseases and how these studies are contributing to a broader understanding of sudden death in the community.

## Introduction

Sudden cardiac death is typically defined as an unexpected natural death from a cardiac cause within 1 hour of the onset of symptoms ^1^. Estimating the prevalence of sudden cardiac death is difficult but is variously estimated as up to half of heart-related deaths or up to 10% of all deaths in our community. Perhaps the most pertinent word in the definition of sudden cardiac death is “unexpected”, which implies that the person’s heart function up until that point was at least reasonable if not perfectly normal. As Jim Forrester so eloquently describes in his recent book, “The Heart Healers: The Misfits, Mavericks, and Rebels Who Created the Greatest Medical Breakthrough of Our Lives”, it cuts short the lives of people with “hearts too good to die” ^2^.

In cases of sudden cardiac arrest, death will occur within a few minutes unless appropriate resuscitation measures are implemented. Despite recent improvements, as a result of factors such as increased access to public defibrillation devices for example ^3^, the overall survival to discharge following an out-of-hospital cardiac arrest is still very low: 10.4% in the latest figures ^4^. There remains an urgent need to improve community-based cardiopulmonary resuscitation ^5^. However, most research effort in this field has been aimed at identifying patients at risk so that preventative measures can be implemented before a cardiac arrest occurs.

Everybody who suffers from a sudden cardiac arrest has an underlying heart condition of some sort. Thus, the incidence of sudden death is roughly correlated with the incidence and severity of the underlying heart disease. In the young (<35 years), inherited heart diseases, including both the primary arrhythmia syndromes and the cardiomyopathies, are the major cause of sudden cardiac death. In the middle age group (35–65), the focus shifts to early onset ischaemic heart disease. In the older age group (>65 years), chronic heart disease, often in combination with other chronic conditions such as type 2 diabetes, becomes increasingly important. Of these three groups, understanding the underlying mechanisms and preventing sudden cardiac death associated with inherited heart diseases is an area of particular interest for two major reasons. First, it usually manifests in the young and so has a particularly devastating effect on families and the community. In this context, establishing a definitive genetic diagnosis can greatly enhance our ability to detect other family members at risk ^6^. Second, because these conditions have relatively simple underlying causes, i.e. a single genetic defect ^7^, it is thought that they will be more tractable to understanding the underlying mechanism of disease and thus provide proof of principle for the development of techniques and technologies applicable in more common acquired arrhythmia syndromes. For the purpose of this brief review into recent advances in the understanding and prevention of sudden cardiac death, we will focus on sudden death in the young (<35 years). Rather than presenting a comprehensive review, we have selected what we believe to be some of the most significant papers of recent years and put them into context of research in the field.

## Epidemiology of sudden death in the young

Our knowledge of rare or uncommon inherited disorders has been greatly facilitated by the collection of large cohorts of well-phenotyped patients, as well as their families, in referral centres such as the international long-QT syndrome (LQTS) registry ^8^. Such registries have provided, and continue to provide, significant insights into the causes and risk factors of disease and have led to the development of algorithms for risk stratification in these cohorts ^9^. Such registries, however, do not necessarily reflect the incidence of the disease in the general community.

The traditional method of establishing the cause of unexpected cardiac deaths has been by post-mortem. However, as post-mortems are not compulsory for all deaths, such studies can also be limited in estimating population incidences. Two recent large autopsy series illustrate the problems that this can create. In a 10-year study of 2,661 consecutive autopsies in Oulu, where autopsy rates are high, Hookana *et al*. ^10^ reported that in young people (0–39 years old) a structurally normal heart was found in only 4% of cases and features consistent with classical genetic cardiomyopathies (dilated, hypertrophic, arrhythmogenic right ventricular) were found in only 15%. The most common abnormalities noted were idiopathic interstitial fibrosis (28%) and cardiomyopathy related to obesity (26%). Conversely, in a recent study of 357 consecutive cases of athletes who died suddenly and were referred for specialist cardiac post-mortem examination, Finocchiaro *et al*. ^11^ found that in cases <35 years of age, 48% had structurally normal hearts, i.e. more than 10 times higher than the rate reported in the Finnish study. However, similar to the Finnish study, Finocchiaro *et al*. ^11^ noted a significant number of young cases with idiopathic fibrosis or hypertrophy (13%). The later UK-based study, however, is likely to have had a significant referral bias, as there was no mandate for the treating physician or local pathologist to refer cases if they were already confident of the cause of death. Nevertheless, both of these studies highlight a probably underappreciated cause of sudden death in the young, i.e. idiopathic interstitial fibrosis, which is certainly worthy of further investigation.

In this context, the recent study from Bagnall and colleagues ^12^ represents a significant advance in our knowledge of the incidence of sudden cardiac death in the young (defined as 1–35 years in their study). The 3-year study was a prospective, population-based, clinical and genetic study that covered a population of 12.59 million young people in Australia and New Zealand. In total, 490 cases of sudden cardiac death were identified, representing an incidence of 1.3/100,000 per annum, which is slightly lower than previous estimates based on retrospective analyses ^13^. In 292 of the 490 cases, the cause of sudden cardiac death was established at autopsy. The commonest causes of autopsy-explained sudden cardiac death were coronary artery disease (especially in the 30–35 year age group) and cardiomyopathies. Autopsy-negative cases were most common in the 16–20 year age group. In 113 of the 198 autopsy-negative cases, a “molecular autopsy” was performed. A genetic cause of death was established in 27% of these 113 individuals. This is 27% of patients who would not have been diagnosed in previous decades and enabled follow up screening of family members. Nevertheless, the fact that 73% of unexplained cases remain undiagnosed indicates that there is still much to be learned about the causes of sudden cardiac death in the young.

## Causes of sudden cardiac death in the young

In recent years, the advent of next-generation sequencing technologies has greatly facilitated our ability to determine the causes of sudden cardiac death ^6^. These technologies have permitted the discovery of more difficult cases where inheritance is not Mendelian, such as somatic mosaicism. For example, Priest and colleagues used single cell sequencing from a large number of mononuclear cells within a single blood sample to demonstrate that early somatic mosaicism can cause LQTS ^14^. Further, they estimated that this may occur in as many as 1:2,000 cases of LQTS (4/7,500 cases referred for commercial gene panel sequencing). Making a diagnosis of somatic mosaicism, as opposed to germline transmission, is of course very important from a family planning perspective. Another important feature of the study was the use of computational modelling to analyse how incorporating abnormal sodium channel function in only 20% of cells, either distributed randomly or clustered, was sufficient to explain the clinical features of the syndrome in this case. Indeed, computational approaches are becoming an increasingly important tool for investigating how defects at the molecular level can result in the emergent phenotypes observed at the clinical level ^15, 16^. Computational cardiology promises to be an area of much greater activity in years to come ^17^.

## Genotype–phenotype relationships in inherited arrhythmia syndromes

The explosion in whole exome ^18^ and whole genome ^19^ sequencing has greatly enhanced our ability to detect putative loss-of-function mutations in genes associated with sudden cardiac death ^20^. Most of these mutations, however, are private to one family and so determining whether they are indeed the cause of disease or a mere bystander is problematic. To illustrate the potential magnitude of this problem, van Driest and colleagues ^21^ investigated how commonly potential loss-of-function mutations occurred in a prospective cohort study that included 2,022 individuals recruited for non-antiarrhythmic drug exposure phenotypes. Forty-two variants in *KCNH2* or *SCN5A*, identified in 63 participants, were designated as potentially pathogenic, but only two of the subjects had a clearly prolonged QT interval (>500 ms). The major message from this study was that one must be cautious about the interpretation of the pathogenic significance of incidental genetic findings in patients with no overt phenotype. A corollary of this conclusion is that we need to develop more robust, higher-throughput assays to assess the impact of mutations on protein function either in heterologous expression systems (see e.g. 22) or in human induced pluripotent stem cell (iPSC)-derived cardiomyocytes where the impact of mutations can be characterised in a human and cardiac-relevant system ^23^ (discussed in more detail below). It is hoped that such functional assays can be combined with traditional bioinformatics tools, such as sequence conservation and sidechain biochemical properties, to develop algorithms with better predictive accuracy for assessing the pathogenicity of mutations ^24^. A second very important message to emerge from the van Driest study is just how valuable the combination of large-scale electronic health record databases and genomic data can be. This is exemplified by the numerous studies arising from the electronic MEdical Records & GEnomics (eMERGE) network, which was established by the National Human Genome Research Institute (NHGRI) of the National Institutes of Health (NIH) in 2007 ^25^. This initiative has also spawned a new field of research called phenome-wide association studies (PWAS). Rather than starting with a disease and looking for genetic variants associated with that disease (i.e. genome-wide association studies [GWAS]), one can now collect patients with variants in a given gene and look to see what phenotypes are most commonly associated with those genetic variants ^26^.

## Understanding disease mechanisms

Our improved understanding of the molecular and cellular basis of cardiac electrical activity has greatly facilitated the development of multi-scale computational models of the heart ^27^. Beyond modelling the impact of single gene defects ^15, 28^, these models are now being combined with sophisticated statistical methods to investigate how multiple genetic hits, with or without environmental insults, can modify the impact of a primary genetic mutation ^29, 30^ to help us understand the variable presentation of disease genes in the population.

Of potentially equal, and complementary, impact has been the development of cellular cardiomyocyte models using patient-derived iPSCs ^23^. iPSC technology enables direct analysis of the impact of a mutation in cellular and simple tissue-level context ^20^. It should also be possible to study any given mutation in different genetic backgrounds (e.g. using iPSCs derived from different patients with the same disease gene), so gaining insights into population-level variability in phenotypes. However, there is still considerable work that needs to be done to develop robust methods for the generation of iPSC-derived cardiac myocytes so that one can be confident that changes observed in any given genetic background can be attributed to that genetic background and not some confounding uncontrolled factor in the iPSC differentiation process. Studying the impact of mutations in a cellular context also allows the analysis of drug therapies. For example, Sala *et al*. recently showed that a drug that allosterically modulates *I*_Kr_ activity can ameliorate the electrical impact of *HERG* and *KCNQ1* mutations in iPSC-derived cardiac myocytes ^31^. It has also been proposed that these models may be used for “clinical trials in a dish” ^32^ to facilitate patient-specific or so-called precision medicine initiatives. Another recent development that promises to greatly enhance the utility of iPSCs in examining disease phenotypes *in vitro* is the development of genome editing approaches such as CRISPR-Cas9, which permits the introduction (or removal) of any mutation in the genome. Using this technology, a range of mutants corresponding to a particular disease can be inserted into isogenic backgrounds to evaluate disease severity on a true like-for-like basis. Alternatively, the same primary disease gene can be inserted into a range of genetically diverse backgrounds to assess population variability in disease presentation *in vitro*. For an excellent recent review on the structure and mechanism of CRISPR/Cas9 activity, see 33.

## Conclusion

Over the next few years, we expect that deeper phenotyping integrated with more sophisticated and larger electronic health record databases will accelerate discovery in the biomedical sciences and this will ultimately pave the way for more accurate diagnoses with greater mechanistic insights. There is also hope for the development of patient-tailored treatments based on mutation-specific pharmacology or insights gained from patient-specific iPSC-derived cardiomyocytes. In the case of sudden cardiac arrest, this should permit the implementation of preventative strategies at an earlier stage.
